# Blocking GSDME-mediated pyroptosis in renal tubular epithelial cells alleviates disease activity in lupus mice

**DOI:** 10.1038/s41420-022-00848-2

**Published:** 2022-03-12

**Authors:** Guihu Luo, Yi He, Fangyuan Yang, Zeqing Zhai, Jiaochan Han, Wenchao Xu, Jialin Zhang, Lili Zhuang, Yanan Zhang, Yehao Li, Rui Song, Xiaoqing Luo, Jianheng Liang, Erwei Sun

**Affiliations:** 1 The Third Affiliated Hospital of Southern Medical University, Department of Rheumatology and Immunology, Guangzhou, China; 2 Shunde Hospital of Southern Medical University, Department of Rheumatology and Immunology, Foshan, China

**Keywords:** Systemic lupus erythematosus, Cell death and immune response

## Abstract

An increase in apoptosis and/or defects in the clearance of apoptotic cells resulting in massive secondary necrosis have been recognized as the main causes of systemic lupus erythematosus (SLE). Recent findings have revealed that gasdermin E (GSDME)-mediated pyroptosis is a mechanism associated with secondary necrosis. We aimed to investigate the effects of GSDME-mediated pyroptosis on disease activity in lupus mice. In vivo, high levels of GSDME expression were observed in the renal tubules of pristane-induced lupus (PIL) mice and SLE patients. In lupus mice, GSDME knockout or SP600125 administration effectively ameliorated lupus-like features by inhibiting GSDME-mediated renal tubular epithelial cell pyroptosis. In vitro, treatment with tumour necrosis factor-α (TNF-α) plus cycloheximide (CHX) or SLE sera induced HK2 cells to undergo pyroptosis in a caspase-3- and GSDME-dependent manner. Likewise, SP600125 significantly reduced GSDME expression and decreased pyroptosis in HK2 cells. GSDME-mediated pyroptosis may be associated with SLE pathogenesis, and targeting GSDME may be a potential strategy for treating SLE.

## Introduction

Systemic lupus erythematosus (SLE) is a complex autoimmune disease involving multiple organs [1]. The overall incidence of SLE ranges from 30.0 to 37.6 cases per 100,000 per year in China [2]. To date, extensive studies have been conducted on the pathogenesis and treatment of SLE, but the molecular mechanism of SLE pathogenesis remains unclear. An increase in apoptosis and defective clearance of apoptotic cells are observed in SLE, and these apoptotic cells have an increased likelihood of progressing to secondary necrosis. Many studies have demonstrated that secondary necrosis of apoptotic cells plays an important role in the pathogenesis and development of SLE [3–5].

Cell death plays an important role in maintaining homoeostasis and the immune response, and different types of cell death have different effects on the immune response. More than 10 years ago, our research group proposed the cell death recognition model [6, 7], which emphasized the different patterns of cell death that determine the outcomes of immune responses. Necrotic cells initiate the secretion of proinflammatory cytokines to activate the immune response, while apoptotic cells induce immune tolerance by inducing anti-inflammatory cytokine production. Although apoptotic cells themselves induce immune tolerance, secondary necrosis occurs when apoptotic cells are not cleared in a timely and effective manner, which can significantly promote inflammatory factor release, enhance the immune response, and participate in the pathogenesis of SLE [5]. Therefore, preventing the secondary necrosis of apoptotic cells and maintaining cells in the apoptotic state may be one strategy to prevent autoimmune diseases such as SLE.

Secondary necrosis after apoptosis was once considered to be a passive and uncontrollable process of cell death due to the exhaustion of cellular energy and a failure to maintain osmotic pressure [8, 9]. However, recent findings revealed that secondary necrosis after apoptosis was also a programmed process called pyroptosis that was mediated by gasdermin E (GSDME) [10, 11]. In GSDME-high cells, the previously presumed key apoptosis inducer, activated caspase-3, cleaves GSDME to generate the GSDME-N domain. Subsequently, the GSDME-N domain assembles in the cell membrane to form pores, thereby resulting in secondary necrosis. This finding is very encouraging because it suggests the possibility of preventing the secondary necrosis of apoptotic cells and raises new hope for the treatment of SLE.

C-Jun N-terminal kinases (JNKs) are mitogen-activated protein kinases (MAPKs) that can be activated by different stress stimuli and have various regulatory roles [12]. JNKs are involved in the development and progression of inflammatory diseases [13]. Importantly, studies have shown that increased JNK activation is associated with disease activity and organ injury in patients with SLE [14]. Interestingly, one study showed that the JNK inhibitor SP600125 could prevent GSDME-mediated cell death [15], suggesting that JNK may be a target for regulating GSDME.

Therefore, this study aimed to investigate whether inhibiting JNK could reduce GSDME-mediated pyroptosis and thus alleviate the disease condition of pristane-induced lupus (PIL) mice.

## Results

### Increased GSDME expression in the kidneys of SLE patients and PIL mice

Lupus nephritis, which is the main clinical manifestation of SLE [16], develops in most SLE patients within 5 years of diagnosis [17]. Therefore, we examined kidney specimens from SLE patients and PIL mice.

**Fig. 1:**
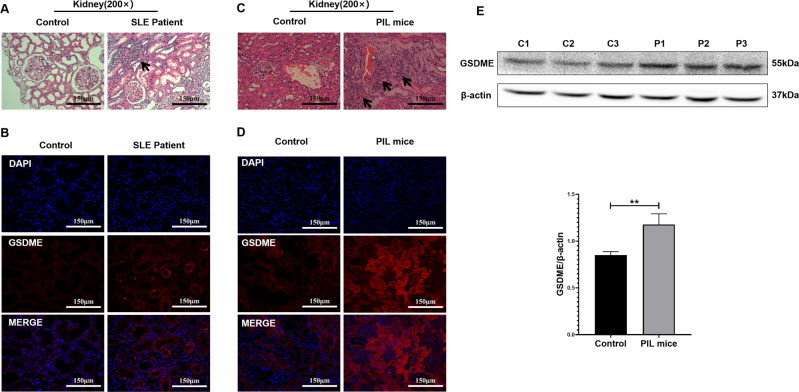
GSDME was highly expressed in the renal tubular cells of SLE patients and PIL mice. **A** H&E staining showing renal pathological changes in control (non-lupus nephritis) and SLE patients (lupus nephritis type IV-V) (Table 1). **B** Immunofluorescence analysis of the expression of GSDME in kidney specimens from controls and SLE patients. **C** H&E staining showing renal pathological changes and **D** immunofluorescence analysis of GSDME expression in control and PIL mouse kidneys. **E** Western blot (top) and quantitative analysis (bottom) of the expression of GSDME in control and PIL mouse kidneys. The data are shown as the mean ± SD, **p < 0.01.

In the kidneys of SLE patients and PIL mice, the renal tubulointerstitium was infiltrated by a large number of inflammatory cells, and there was renal tubule disruption, glomerular atrophy, and interstitial fibrosis (Fig. 1A, C). Importantly, GSDME was highly expressed in renal tubules (Fig. 1B) in SLE patients and PIL mice (Fig. 1D, E).

### Tumour necrosis factor-α (TNF-α) plus cycloheximide (CHX) or lupus patient sera induced pyroptosis in HK2 cells

GSDME plays a key role in pyroptosis [10, 11]. Given that high levels of GSDME were found in the renal tubules of lupus kidneys, we next investigated whether renal tubule epithelial cells develop GSDME-mediated pyroptosis. In vitro, human tubular epithelial cells (HK2 cells) were treated with the apoptosis inducers TNF-α and CHX [11], and pyroptosis was examined.

**Fig. 2:**
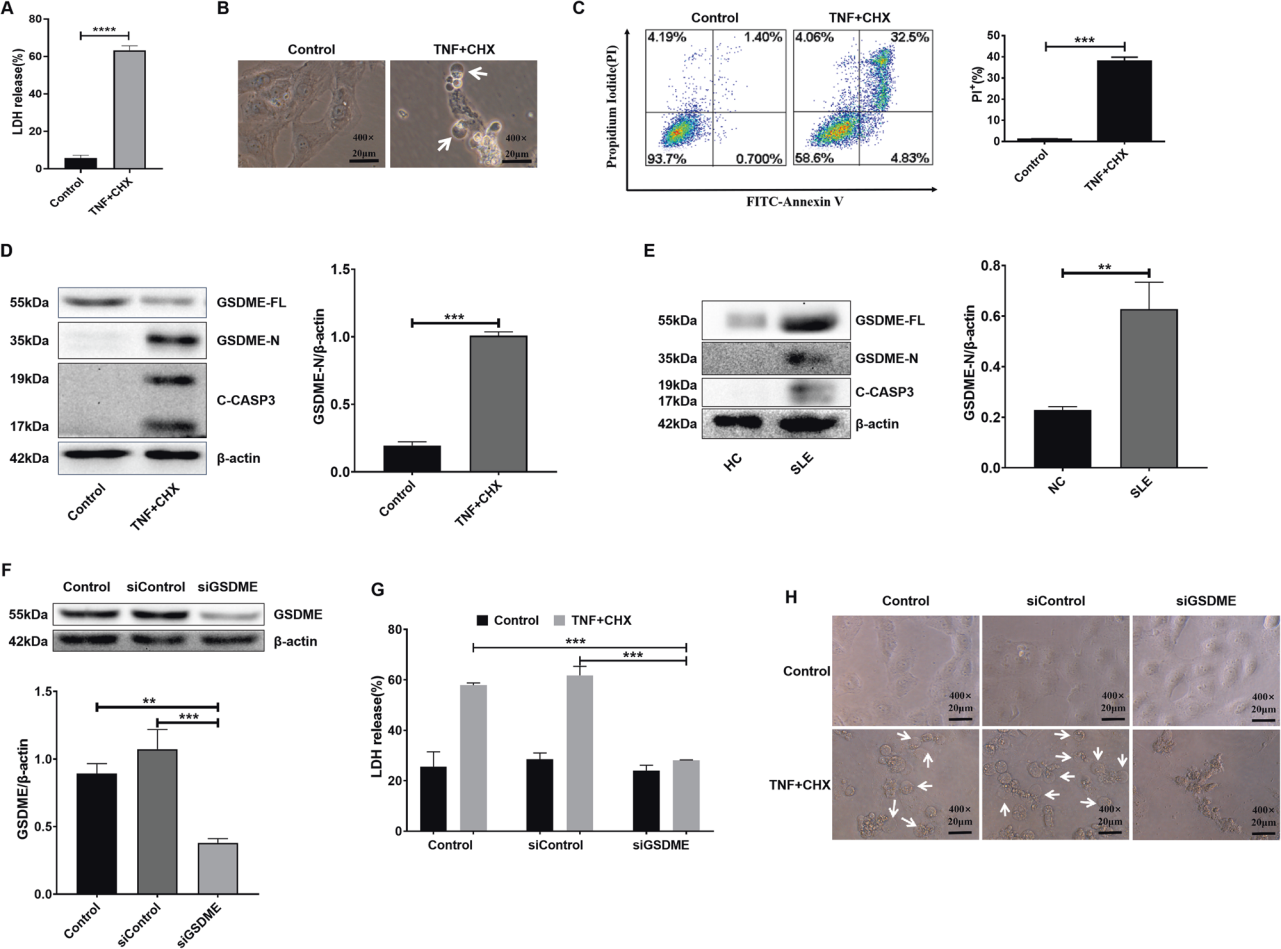
TNF-α+CHX or lupus patient sera induced GSDME-mediated pyroptosis in HK2 cells. **A** HK2 cells were treated with TNF-α and CHX for 12 h, and cell viability was measured by examining LDH release. **B** Phase-contrast imaging of HK2 cells after TNF-α and CHX treatment. **C** Flow cytometric analysis of PI- and Annexin V-FITC-stained cells. **D** and **E** Western blot (left) and quantitative analysis (right) of the expression of GSDME (*n* = 3) in HK2 cells treated with TNF-α+CHX and sera (from lupus patients or healthy controls). **F** HK cells were transfected with NC- or GSDME-siRNA. **G** LDH release and **H** phase-contrast images of HK2 cells were examined after TNF-α and CHX treatment. Arrows, pyroptotic cells. The data are shown as the mean ± SD, **p < 0.01, ***p < 0.001, ****p < 0.0001.

After TNF-α plus CHX stimulation, the number of necrotic HK2 cells was significantly increased (Fig. 2A, C), and dying cells showed evident swelling with characteristic large bubbles protruding from the plasma membrane (Fig. 2B). Moreover, TNF-α- and CHX-stimulated HK2 cells showed elevated levels of activated caspase-3 and GSDME-N (Fig. 2D). These results confirmed that TNF-α and CHX induced GSDME-mediated pyroptosis in HK2 cells, similar to the findings of previous studies [10, 11].

**Fig. 3:**
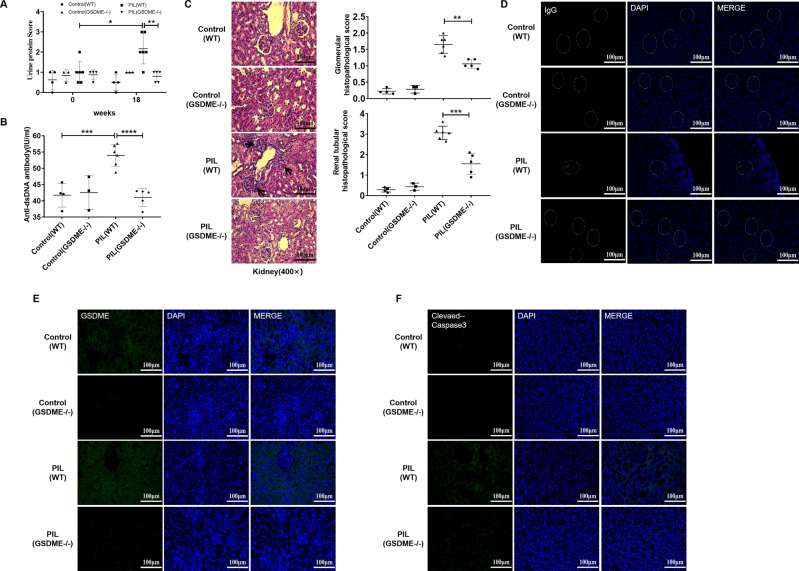
GSDME knockout ameliorated SLE pathogenesis in PIL mice. **A** Albustix test papers were used to determine urinary protein levels. **B** The levels of anti-dsDNA antibodies were examined by ELISA. **C** Representative H&E staining of glomerular and renal tubular lesions in kidneys is shown (left). Austin scores of kidneys were determined (right). **D** Immunofluorescence analysis showing IgG deposition in the kidney. **E**, **F** Immunofluorescence staining of GSDME and cleaved caspase-3 in each group of mouse kidneys. The data are shown as the mean ± SD, *p < 0.05, **p < 0.01, ***p < 0.001.

Because GSDME expression is increased in the renal tubular epithelial cells of lupus patients, it is important to know the mechanisms underlying the increase in GSDME expression. Thus, HK2 cells were stimulated with from the sera of lupus patients, and GSDME expression was examined. Serum was collected from lupus patients or healthy controls (Table 2) and then used to prepare 10% cell culture medium. HK2 cells were cultured in the prepared medium for 72 h. Interestingly, we found that the serum of lupus patients significantly enhanced the expression of GSDME and pyroptosis (Fig. 3E).

**Table 1:** Data of patients with lupus nephritis and controls. Renal specimens were obtained from the pathology department of the hospital.

	Lupus nephritis			Non-lupus nephritis			
Name	LJP	QYZ	DMM	LC	LG	CCS	LDW
Sex	Male	Female	Female	Female	Male	Male	Male
Age	47	49	30	42	38	27	44
WBCs(×10^9/L)	4.3	3.6	3.7	6.5	7.7	6.7	11
RBCs(×10^12/L)	4.03	3.54	3.56	3.96	5.18	5.12	7.15
HGB(g/L)	120	100	98	119	153	108	144
PLTs (×10^12/L)	241	271		226	194	254	257
Urine leucocyte	1+	2+	Negative	3+	Negative	1+	Negative
Urine erythrocyte	3+	Negative	3+	Negative	Negative	3+	2+
Urine protein	3+	3+	3+	2+	3+	1+	2+
24-hour proteinuria (mg)	6301.96	2697.04	1284.69	1256.65	982.8	875.12	1582.68
ALT(U/L)	14	5	11	11	45	13	17
AST(U/L)	29	13	14	12	29	22	20
Albumin(g/L)	23.4	14.4	26.3	36.2	45.7	17.6	40.9
Creatinine(umol/L)	107	335	121	72	81	75	163
BUN(mmol/L)	6.71	14.27	9.79	6.27	6.37	4.06	8.07
Globulin(g/L)	25.7	21.1	25.5	24.1	35.3	16.2	25.5
IgG (g/L)	8.79	13.51	9.18	8.67	9.2	<2.42	9.07
IgA (g/L)	0.6	2.17	4.19	1.99	2.95	1.04	0.51
IgM (g/L)	3.17	2.94	1.19	1.59	0.39	1.17	3.45
C3 (g/L)	0.42	0.64	0.22	1.03	1.39	1.21	1.07
C4 (g/L)	0.07	0.32	0.02	0.22	0.18	0.2	0.31
ANA	Positive 1:80 (spotted type)	Positive 1:160 (spotted type)	Positive 1:320 (homogeneous type)	Negative	Negative	Negative	Negative
Anti-dsDNA antibody	Positive	Negative	Negative	Negative	Negative	Negative	Negative
Pathologic diagnosis	Lupus nephritis type III	Lupus nephritis type III+V	Lupus nephritis type IV-V	IgA nephropathy	Diabetic nephropathy	Membranous nephropathy	IgA nephropathy Lee’s grade III
SLEDAI score	16	14	17	None	None	None	None

Then, siRNA was used to silence GSDME expression (Fig. 2F), and GSDME-mediated pyroptosis was significantly reduced (Fig. 2G, H).

### GSDME knockout ameliorated SLE pathogenesis in PIL mice

To further investigate the role of GSDME in lupus pathogenesis, we prepared *GSDME*−/− mice (C57BL/6-GSDME^tm1cyagen^ mice). The mice were intraperitoneally administered pristane and developed autoantibodies and clinical manifestations similar to those of SLE patients [18, 19]. One study demonstrated that pristane increased apoptosis, induced autoantigens, and initiated an immune response that led to the development of lupus-like autoimmunity [20]. After intraperitoneal injection of pristane, *GSDME*−/− mice showed decreased proteinuria (Fig. 3A), reduced autoantibody levels (Fig. 3B), and improvements in kidney pathology (Fig. 3C). Histological analysis of kidney sections was performed to determine whether the reduction in lupus-specific autoantibody production in *GSDME*−/− mice was accompanied by a reduction in glomerular IgG deposition. As shown in Fig. 3D, *GSDME*−/− mice had reduced glomerular IgG deposition. Furthermore, we found that the expression of cleaved caspase-3 was decreased in *GSDME*−/− mice (Fig. 3F), accompanied by a decrease in GSDME expression (Fig. 3E). Thus, *GSDME*−/− mice were at least partially protected against pristane-induced renal disease through the inhibition of pyroptosis.

### SP600125 inhibited GSDME-mediated pyroptosis

**Fig. 4:**
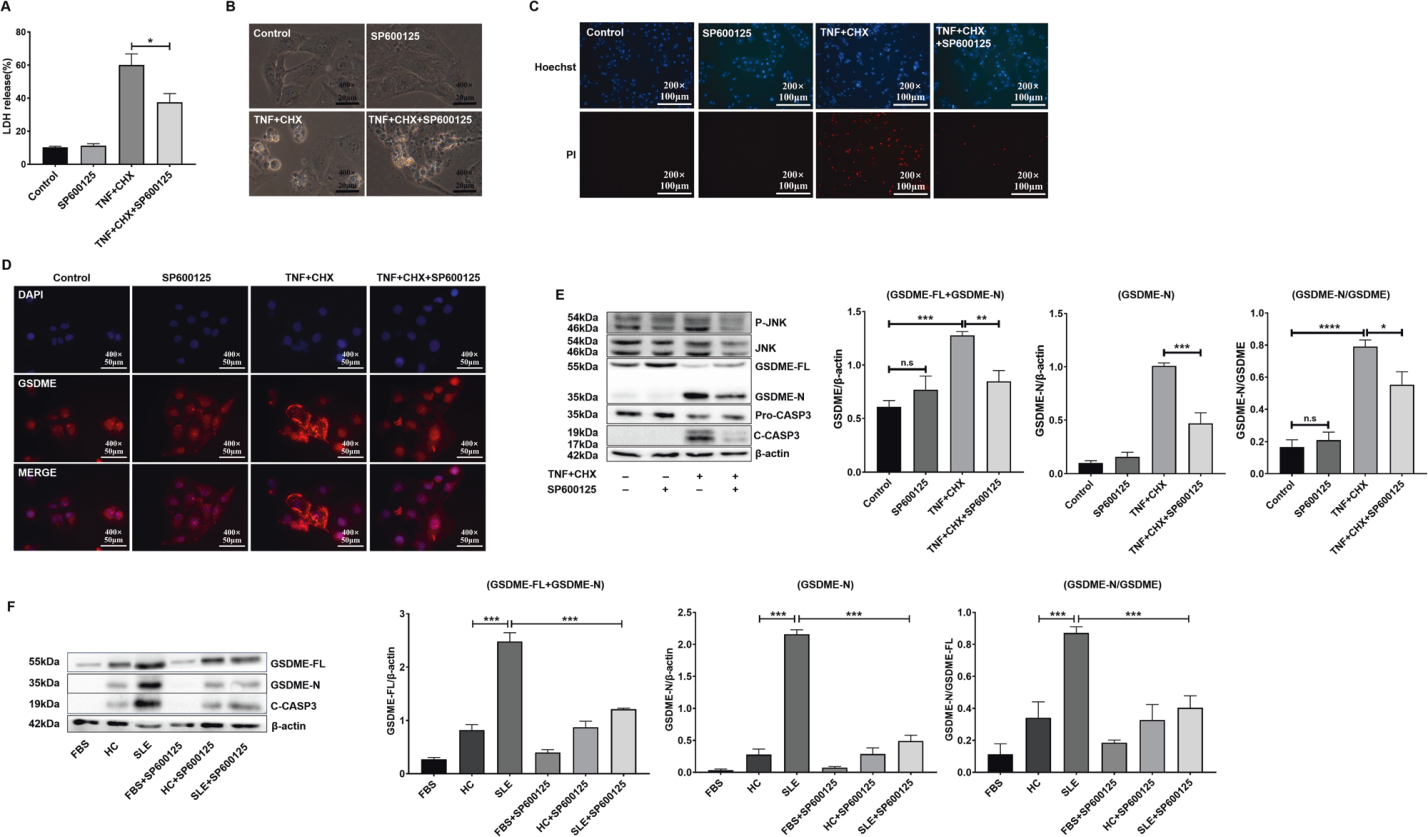
SP600125 inhibited GSDME-mediated pyroptosis in HK cells. HK2 cells were pretreated with SP600125 for 1 h and then exposed to TNF-α plus CHX for 12 h. **A** The levels of LDH were measured. **B** Phase-contrast images of HK2 cells were collected. **C** HK2 cells were stained with Hoechst (blue) and PI (red). **D** Immunofluorescence staining of GSDME (red). **E** The expression of GSDME-FL and GSDME-N was measured by Western blotting. **F** The sera of lupus patients (*n* = 5) or healthy controls (*n* = 3) were mixed together and then added to the medium to prepare 10% cell culture medium. HK2 cells were pretreated with SP600125 for 1 h and then exposed to the serum of lupus patients or normal controls, followed by Western blot and quantitative analysis of the expression of GSDME. The data are shown as the mean ± SD, *p < 0.05, **p < 0.01, ***p < 0.001.

JNK is a MAPK that is involved in the pathological process of inflammation. A previous study reported that the JNK inhibitor SP600125 blocked GSDME-mediated cell death [21]. Next, we investigated the effect of SP600125 on GSDME-mediated pyroptosis in HK2 cells. We found that SP600125 significantly decreased GSDME-mediated pyroptosis, as indicated by decreased lactate dehydrogenase (LDH) release (Fig. 4A), diminished cell swelling (Fig. 4B), and reduced numbers of propidium iodide (PI)-positive cells (Fig. 4C). Interestingly, GSDME expression increased after TNF-α plus CHX treatment but decreased significantly when SP600125 was applied (Fig. 4D). Total GSDME includes full-length GSDME (GSDME-FL) and the N-terminus of GSDME (GSDME-N). We found that SP600125 inhibited the production of total GSDME and GSDME-N (Fig. 4E), suggesting that SP600125 can inhibit both the expression and activity of GSDME. These results confirmed that SP600125 significantly blocked pyroptosis in HK2 cells by reducing GSDME-mediated cell signalling. Likewise, SP600125 treatment reduced serum-induced GSDME expression and pyroptosis in HK2 cells (Fig. 4F).

### SP600125 effectively attenuated disease activity in PIL mice

**Fig. 5:**
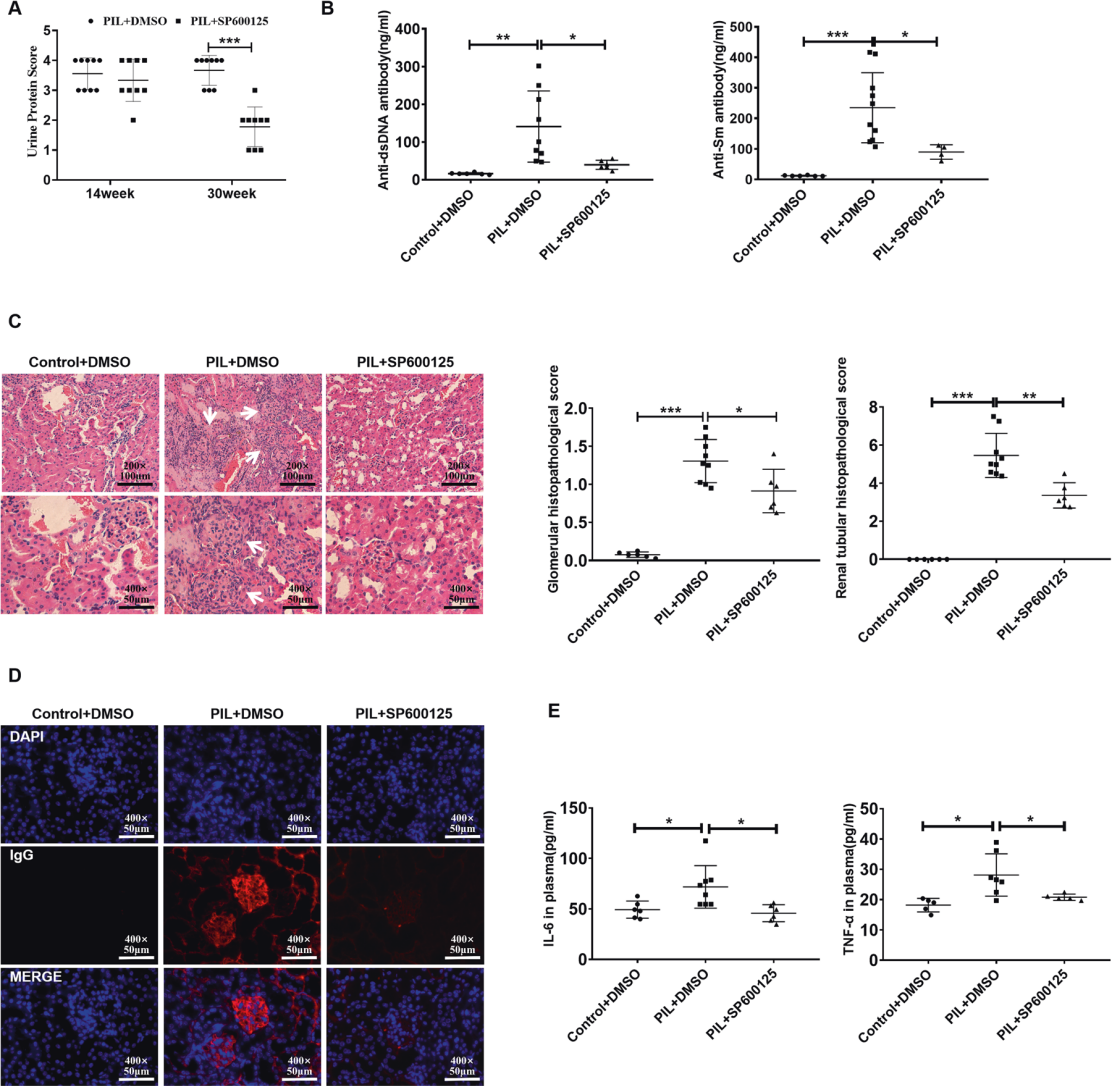
SP600125 effectively attenuated disease activity in PIL mice. **A** Albustix test papers were used to determine urinary protein levels. Urine protein scores were measured once every two weeks. **B**, **E** The levels of autoantibodies (anti-dsDNA and anti-Sm antibodies) and cytokines (IL-6 and TNF-α) were examined by ELISA. **C** Representative H&E staining of glomerular and renal tubular lesions in kidneys is shown (left). Austin scores of kidneys were determined (right). **D** Immunofluorescence analysis showing IgG deposition in the kidney. The data are shown as the mean ± SD, *p < 0.05, **p < 0.01, ***p < 0.001.

To determine whether SP600125 could attenuate SLE activity, we treated PIL mice with SP600125 for 16 weeks. Importantly, SP600125 treatment not only reduced proteinuria (Fig. 5A) but also decreased the levels of anti-dsDNA and anti-Sm antibodies (Fig. 5B). In addition, haematoxylin and eosin (H&E) staining of the kidney revealed that SP600125 treatment alleviated renal injury (Fig. 5C), decreased IgG deposition (Fig. 5D), and decreased plasma IL-6 and TNF-α levels (Fig. 5E). Taken together, these findings suggested that SP600125 treatment could alleviate lupus-like features in PIL mice.

### SP600125 inhibited GSDME-mediated pyroptosis in PIL mice

**Fig. 6:**
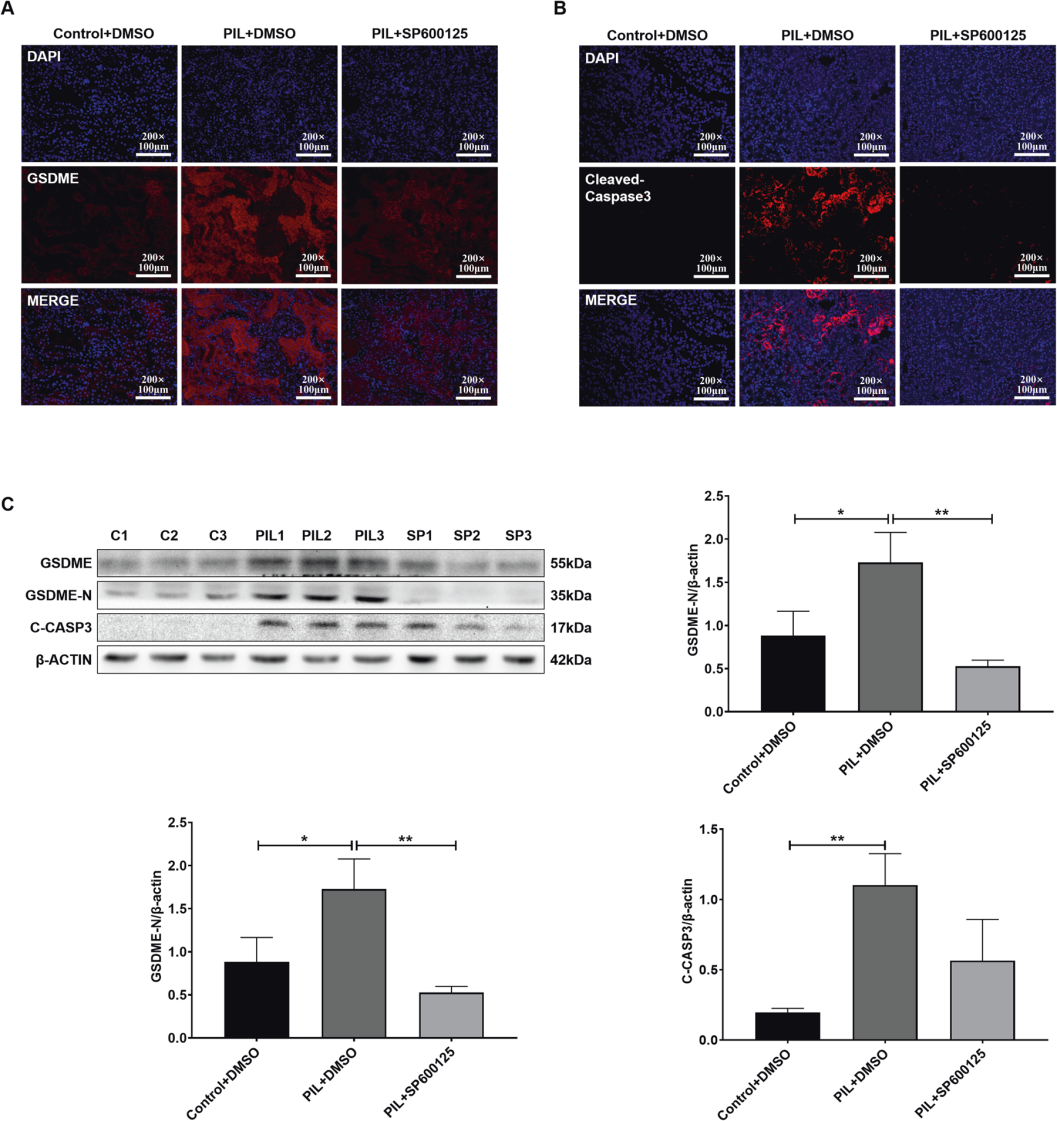
SP600125 inhibited GSDME-mediated pyroptosis in PIL mice. **A**, **B** Immunofluorescence staining of GSDME and cleaved caspase-3 in each group of mouse kidneys. **C** Western blot and quantitative analysis of the expression of GSDME and cleaved caspase-3. The data are shown as the mean ± SD, *p < 0.05,**p < 0.01.

Compared with that in the control group, the expression of GSDME in renal tubular epithelial cells in the PIL group was significantly increased (Fig. 6A), as was the expression of cleaved caspase-3 (Fig. 6B). Importantly, lupus-prone mice that were treated with SP600125 exhibited reduced protein expression levels of GSDME and cleaved caspase-3 (Fig. 6A, B). In particular, abundant levels of GSDME-N in the kidney were easily detected in vehicle-treated PIL mice but were rarely found in SP600125-treated PIL mice (Fig. 6C). Based on these results, SP600125 inhibited GSDME-mediated pyroptosis in the kidney.

## Discussion

SLE is an autoimmune disease that is characterized by the presence of nuclear autoantibodies and involves multiple organs [22]. Although the survival rate of SLE patients has improved in the past decade [23], recent advances have not reduced mortality or the development of end-stage lupus nephritis [24, 25], suggesting that further study of the pathogenesis of SLE is of great clinical value.

In this study, high levels of GSDME were observed in the renal tubules of SLE patients. Moreover, the expression of GSDME and cleaved caspase-3 in the renal tubules of PIL mice was also increased, suggesting that GSDME-mediated pyroptosis may play an important role in the pathogenesis and development of SLE (Figs. 1 and 6).

Subsequently, renal tubular epithelial cells (HK2 cells) were selected as the study objects to verify the mechanism of GSDME-mediated pyroptosis. HK2 cells were stimulated with TNF-α plus CHX and exhibited elevated protein expression levels of cleaved caspase-3 and GSDME-N and increases in GSDME-mediated pyroptosis (Fig. 2). Importantly, the sera of SLE patients also induced HK2 cells to undergo GSDME-mediated pyroptosis (Fig. 2E).

To further verify the role of GSDME in lupus, we stimulated *GSDME*−/− mice with pristane. After intraperitoneal injection of pristane, *GSDME*−/− mice exhibited ameliorated SLE pathogenesis, with reduced levels of autoantibodies, decreased proteinuria, and improved renal pathology (Fig. 3). These results showed that GSDME plays an important role in the pathogenesis of lupus.

Interestingly, the JNK inhibitor SP600125 significantly reduced the expression of GSDME and pyroptosis in HK2 cells (Fig. 4). In vivo, SP600125 administration effectively ameliorated lupus-like features in PIL mice (Fig. 5). Importantly, PIL mice treated with SP600125 showed reduced expression levels of GSDME and cleaved caspase-3 (Fig. 6). Based on these results, we believe that SP600125 effectively attenuates disease activity in PIL mice by inhibiting GSDME-mediated pyroptosis.

GSDME, which is also known as deafness, autosomal dominant 5 (DFNA5), was first associated with sensorineural hearing loss in humans [26]. Most deafness-causing mutations in GSDME lead to the deletion of GSDME C-terminal transcription and could induce spontaneous pyroptosis [27]. In addition to hearing loss, GSDME has been linked to many cancers, such as breast [28, 29], hepatocellular [30], gastric [31], and colorectal [32] cancers. In these cancers, GSDME methylation is significantly increased, resulting in epigenetic silencing, which reduces GSDME levels [33]. GSDME can be regulated by p53 because of the presence of p53 binding sites in GSDME intron 1 [33]. Treatment with demethylating agents, such as 5-aza-2′-deoxycytidine, can restore p53-induced GSDME expression [34]. Based on these reports, *GSDME* is considered to be a tumour suppressor gene because GSDME inactivation inhibits its necrotic effects, thus promoting tumour formation.

GSDME has roles in switching the cell death mode from apoptosis to pyroptosis [35]. Caspase-3 specifically cleaves and activates GSDME, resulting in pyroptosis. This phenomenon changes the concept of programmed cell death, as caspase-3 has long been considered to be a marker of apoptosis. In fact, the expression level of GSDME determines the mode of cell death in Caspase-3-activated cells [11]. Cells with high GSDME expression undergo pyroptosis after being stimulated with apoptotic inducers, such as chemotherapy drugs. However, cells lacking sufficient GSDME undergo apoptosis.

GSDME may play a role in chemotherapy-induced caspase-3-dependent cell death due to its ability to regulate apoptosis and pyroptosis [11]. In an in vivo study, *GSDME*^−/−^ mice were protected from chemotherapy drug-induced tissue damage and weight loss [11]. Intraperitoneal injection of cisplatin or 5-FU caused severe small intestinal impairment and immune cell infiltration in *GSDME*^+/+^ mice, whereas these signs of tissue damage were reduced in *GSDME*^−/−^ mice [35]. Moreover, *GSDME*^−/−^ mice showed attenuated lung injury and inflammation in response to cisplatin or bleomycin [35]. These observations confirmed the critical role of GSDME-mediated cell death in promoting inflammation and organ damage. However, the role of GSDME-mediated pyroptosis in lupus has not been reported. Here, we found high levels of GSDME in kidney specimens from SLE patients and lupus-prone mice. We also provided evidence that GSDME-mediated pyroptosis may play an important role in the pathogenesis and development of SLE. Therefore, preventing GSDME-mediated pyroptosis is a new strategy for the treatment of SLE.

JNKs, which are members of the MAPK family, regulate physiological processes such as neuronal function, immune function, and embryonic development by influencing gene expression, cytoskeletal protein dynamics, and cell death/survival pathways [36]. In response to apoptotic stimulation, JNKs regulate the activity of various proapoptotic and antiapoptotic proteins, thus participating in extrinsic and intrinsic apoptotic pathways [37]. Multiple abnormalities in the JNK pathway are involved in several autoimmune diseases, including chronic idiopathic urticaria, inflammatory bowel disease, and SLE [38]. In SLE patients, increased JNK activity correlates with disease activity [39] and long-term organ damage [40]. SP600125 is a commonly used highly selective JNK inhibitor. Studies have shown that JNK inhibitors, including SP600125, can significantly alleviate the symptoms of immune diseases [14]. One study showed that the JNK inhibitor SP600125 could block GSDME-mediated cell death [15], but the mechanism was unclear. We found that SP600125 could significantly improve the symptoms of lupus in mice by inhibiting GSDME-mediated pyroptosis in two ways. First, SP600125 can inhibit the activation of caspase-3 and reduce the development of apoptotic cells. Second, SP600125 can reduce the expression and activation of GSDME, which inhibits pyroptosis but initiates apoptotic signalling. Interestingly, other inhibitors of JNK, JNK-in-8, and JNK Inhibitor VIII, as well as SP600125, inhibited GSDME-mediated cell death (Supplementary Fig. S2). In this context, JNK inhibitors may be ideal drugs for the treatment of lupus.

Activated MAPK molecules ultimately activate the transcription factor NF-κB and the subsequent production of inflammatory factors [41]. We then wondered whether SP600125 inhibited GSDME-mediated pyroptosis by blocking the NF-κB pathway. Interestingly, we found that SP600125 inhibited TNF-α+CHX-induced nuclear translocation of p65 in HK2 cells (Supplementary Fig. S1A). Moreover, the NF-κB inhibitor EVP4593 inhibited GSDME-mediated pyroptosis in HK2 cells induced by TNF-α+CHX (Supplementary Fig. S1B). Consistent with these findings, the JNK-NF-κB signalling pathway was shown to be involved in GSDME-mediated pyroptosis in HK2 cells.

One limitation of this study should be noted. SP600125 can only indirectly regulate GSDME expression and activation, and currently, there are no specific GSDME inhibitors. Therefore, our next study will focus on the search for GSDME-specific inhibitors.

In conclusion, our data demonstrated that the JNK inhibitor SP600125 effectively attenuated disease activity in PIL mice by inhibiting GSDME-mediated pyroptosis. This finding highlights the important role of GSDME-mediated pyroptosis in SLE pathogenesis and development and suggests that SP600125 may be a potential drug for treating SLE.

## Materials and methods

### Materials

Rabbit polyclonal antibodies against cleaved Caspase 3 and the N-terminus of GSDME were purchased from Abcam (Catalogue Nos. ab13847 and ab175614, Cambridge, UK). Rabbit monoclonal antibodies against GSDME-N, JNK1+JNK2+JNK3, and phosphorylated JNK1+JNK2+JNK3 (phospho-T183+T183+T221) were also obtained from Abcam (Catalogue Nos. ab215191, ab179461, and ab124956, Cambridge, UK). TNF-α was purchased from InvivoGen (Catalogue No. rcyc-hTNF-α, San Diego, USA). CHX was purchased from Sigma-Aldrich (Catalogue No. 66-81-9, St. Louis, MO, USA). TNF-α and CHX act as inducers of apoptosis [11]. JNK inhibitors (SP600125, JNK-IN-8, and JNK Inhibitor VIII) and an NF-κB inhibitor (EVP4593) were purchased from Selleck (Catalogue No. S1460, Houston, TX, USA). C57BL/6-GSDME^tm1cyagen^ mice were purchased from Cyagen Biosciences Inc. (Contract No.: KOAI180602BX3, Suzhou, China).

### Cell culture

The human tubular epithelial cell line HK2 (Human Kidney-2) was obtained from Bio-Rad Laboratories (Shanghai, China), and the cells were cultured in DMEM/F12 media containing 10% foetal bovine serum (FBS) and 1% penicillin-streptomycin. The cells were stimulated with TNF-α (20 ng/ml) and CHX (10 μg/ml) to induce cell death, with or without pretreatment with SP600125 (30 μM) for 1 h.

### LDH release assay

After TNF-α and CHX stimulation, LDH levels in cell culture supernatants were measured by using an LDH release kit (Promega) according to the manufacturer’s protocol. Supernatant LDH activity is expressed as the percentage of total LDH in the cell lysate.

### Microscopy

To examine the morphological changes in apoptotic or pyroptotic cells, HK2 cells were seeded in six-well plates and subjected to the indicated treatments. Bright field images were captured using an optical inverted microscope (Olympus, Japan). All image data represent at least three random fields of view.

### HK2 cell Hoechst/PI fluorescent staining

HK2 cells were seeded in 24-well plates and treated with TNF-α and CHX. Then, the cells were stained with Hoechst (Beyotime, China) and PI (BD, USA). Images were collected with a fluorescence microscope (Carl Zeiss, Germany).

### Immunofluorescence analysis of GSDME

After stimulation with TNF-α and CHX, the expression of GSDME was examined by immunofluorescence analysis. Cells were fixed with 4% paraformaldehyde, permeabilized with 0.2% Triton X-100, and blocked with 5% FBS. Then, the cells were stained with rabbit anti-GSDME antibodies and incubated with Cy3-conjugated goat anti-rabbit IgG secondary antibodies (Servicebio, China). Finally, the cells were stained with DAPI (Thermo Fisher Scientific, USA). Images were captured and analyzed with a fluorescence microscope (Carl Zeiss, Germany).

### Western blotting

Cells or tissues were lysed in RIPA buffer (Beyotime, China). Cell lysates were fractionated by SDS-polyacrylamide gel electrophoresis (SDS-PAGE) and then transferred to PVDF membranes (Roche, Switzerland). The blots were probed with the appropriate antibodies. The expression of full-length GSDME and the GSDME N-terminus was examined using anti-GSDME antibodies. The data were analyzed by ImageJ software.

### siRNA knockdown

Scrambled siRNA (control) or GSDME-specific siRNA (5′-GCGGTCCTATTTGATGATGAA-3′) was transfected into HK2 cells using Lipofectamine 2000 (Invitrogen, Catalogue No. 11668-027) according to the manufacturer’s protocol. Forty-eight hours later, the transfected cells were treated with TNF-α plus CHX and analyzed as indicated.

### PIL models

As described previously, female BALB/c mice (6–8 weeks old) were intraperitoneally injected with 0.5 ml of pristane (Sigma-Aldrich) [42]. Urine protein was measured at 6 weeks after modelling, and the urine protein score was determined once every two weeks. Albustix test paper was used to examine urinary protein levels. The mice were stimulated to urinate by gentle massage of the abdomen, and urine was collected. Fresh urine was dropped into the reaction area of the test paper, and the results were read within 1 min. The urine protein score was determined by comparing the colour of the reaction area and the standard colour band. If the urine protein score was more than 1 point in two consecutive tests, modelling was considered successful.

PIL mice were divided into three groups: the vehicle-only control group, the PIL group (*n* = 9), and the PIL+SP600125 group (*n* = 6). Six normal mice were treated with vehicle as a control. In the PIL group, the mice received equal amounts of vehicle solution (dimethyl sulfoxide (DMSO) and phosphate-buffered saline (PBS)). In the PIL+SP600125 group, the mice were intraperitoneally injected with SP600125 dissolved in 2% DMSO in PBS at a dose of 30 mg/kg body weight once per day [43]. This study was approved by the Ethics Committee of The Third Affiliated Hospital, Southern Medical University.

### ELISA analysis of plasma autoantibodies and cytokines

Plasma was harvested from the mice and stored at −80 °C. The plasma levels of autoantibodies and cytokines in the mice were measured by ELISA kits (BOSTER, Wuhan, China and NEOBIOSCIENCE, Shenzhen, China).

### Immunofluorescence analysis of GSDME/Caspase-3 p17

Frozen kidney sections were blocked with 5% FBS, stained with rabbit anti-GSDME antibodies (Abcam, Catalogue No. ab215191) and rabbit anti-caspase-3 p17 antibodies (Abcam, Catalogue No. ab13847), and then stained with Cy3-conjugated goat anti-rabbit IgG secondary antibodies (Servicebio, Catalogue No. GB21303). To assess IgG deposition in the kidneys, frozen kidney sections were stained with Alexa Fluor 555-conjugated goat anti-mouse IgG (Abcam, Catalogue No. b150114).

### Assessment of histopathological changes

Renal tissues were fixed with 10% formalin and paraffin-embedded for tissue sectioning. The sections were then stained with H&E. Histopathological changes were examined by pathologists who were blinded to the experimental information. Austin scores of lupus disease activity were determined as described previously [44].

### Statistical analysis

All data are expressed as the mean ± standard deviation, were analyzed by SPSS 20.0 software, and were plotted with GraphPad Prism 7.0. One-way ANOVA was used to compare the means between groups. Differences were considered statistically significant when *p* < 0.05.

**Table 2:** Data of lupus patients and healthy controls. The sera of lupus patients or healthy controls were prepared.

	SLE patients					Health controls		
Name	ZJW	LXL	YXM	DDC	HJW	XD	ZTF	QXY
Sex	Female	Female	Female	Male	Male	Female	Female	Female
Age	23	36	12	28	9	22	21	22
WBCs(×10^9/L)	5.7	4.3	6.5	2.2	2.6	5.1	6.2	7.2
RBCs(×10^12/L)	3.28	3.9	5.06	4.02	3.68	4.4	4.5	5.7
HGB(g/L)	88	119	116	118	104	122	132	144
PLTs (×10^12/L)	265	196	279	143	136	234	243	256
Urine leucocyte	1+	Negative	1+	Negative	Negative	Negative	Negative	Negative
Urine erythrocyte	3+	Negative	Negative	Negative	Negative	Negative	Negative	Negative
Urine protein	1+	Negative	Negative	Negative	Negative	Negative	Negative	Negative
24-hour proteinuria (mg)	820.41	133.65	67.66	387.72	109.4	58.8	66.7	86.1
ALT(U/L)	26	38	18	106	58	22	32	11
AST(U/L)	31	26	22	71	104	23	26	12
Albumin(g/L)	30.9	48.9	40	32.7	33.1	45.1	46.2	42.1
Creatinine(umol/L)	68	116	51	67	43	50	67	70.1
BUN(mmol/L)	3.32	7.01	3.01	4.1	3.43	3.41	3.61	4.56
Globulin(g/L)	38.6	37.2	26.7	21.8	33.1	32.1	30.1	27.6
IgG (g/L)	18.6	19.51	8.75	11.03	20.32	15.4	13.4	13.5
IgA (g/L)	5.57	1.83	1.41	1.28	1.17	3.51	3.61	3.81
IgM (g/L)	1.74	0.88	1.16	0.36	1.19	2.41	1.54	1.81
C3 (g/L)	0.23	0.92	0.93	0.28	0.3	1.55	1.12	1.2
C4 (g/L)	0.02	0.09	0.19	0.04	0.03	0.21	0.33	0.41
ANA	Positive 1:80	Positive 1:160	Positive 1:640	Positive	Positive 1:80	Negative	Negative	Negative
dsDNA(IU/ml)	448.73	26.1	101.3	Positive	Positive	Negative	Negative	Negative
SSA (RU/ml)	68.01	Negative	>400	Negative	Positive	Negative	Negative	Negative
Nuc(RU/ml)	>400	121.64	Negative	Positive	Positive	Negative	Negative	Negative
Ro52(RU/ml)	46.74	47.63	287.36	Negative	Positive	Negative	Negative	Negative
Sm(RU/ml)	Negative	Negative	34.46	Negative	Negative	Negative	Negative	Negative
RNP(RU/ml)	Negative	>400	>400	Negative	Negative	Negative	Negative	Negative
PO(RU/ml)	Negative	Negative	91.79	Negative	Negative	Negative	Negative	Negative
AHA(RU/ml)	Negative	Negative	Negative	Negative	Positive	Negative	Negative	Negative
SLEDAI score	14	10	13	10	10	None	None	None

## Supplementary information

Supplementary figure legends

Supplementary Figure 1

Supplementary Figure 2

Reproducibility checklist

AJE Editing Certificate

## Data Availability

The datasets generated and/or analyzed during the current study are available from the corresponding author upon reasonable request.
