# Supplementary material for: Blocking GSDME-mediated pyroptosis in renal tubular epithelial cells alleviates disease activity in lupus mice
Source: Cell Death Discov. 2022 Mar 12;8:113. doi: 10.1038/s41420-022-00848-2 (PMC8918340; doi:10.1038/s41420-022-00848-2)
Supplement: Supplementary file 1 — Supplementary figure legends [file 41420_2022_848_MOESM1_ESM.docx]

**Supplementary Figure 1. NF-κB signalling pathway was involved in GSDME-mediated pyroptosis of HK2 cells**

1. Immunofluorescence staining showing the nuclear translocation of the p65/NF-κB subunit. (B) HK2 cells were pretreated with EVP4593 (100 nM) for 1 h and then exposed to TNF-α plus CHX for 12 h. The expression of GSDME-FL and GSDME-N was measured by Western blotting.

**Supplementary Figure 2. JNK-in-8 or JNK Inhibitor VIII inhibited GSDME-mediated cell death**

HK2 cells were pretreated with JNK-in-8 or JNK Inhibitor VIII for 1 h and then exposed to TNF-α plus CHX, followed by Western blot and quantitative analysis of the expression of GSDME.
