# Supplementary figures and images for: Blocking GSDME-mediated pyroptosis in renal tubular epithelial cells alleviates disease activity in lupus mice
Source: Cell Death Discov. 2022 Mar 12;8:113. doi: 10.1038/s41420-022-00848-2 (PMC8918340; doi:10.1038/s41420-022-00848-2)

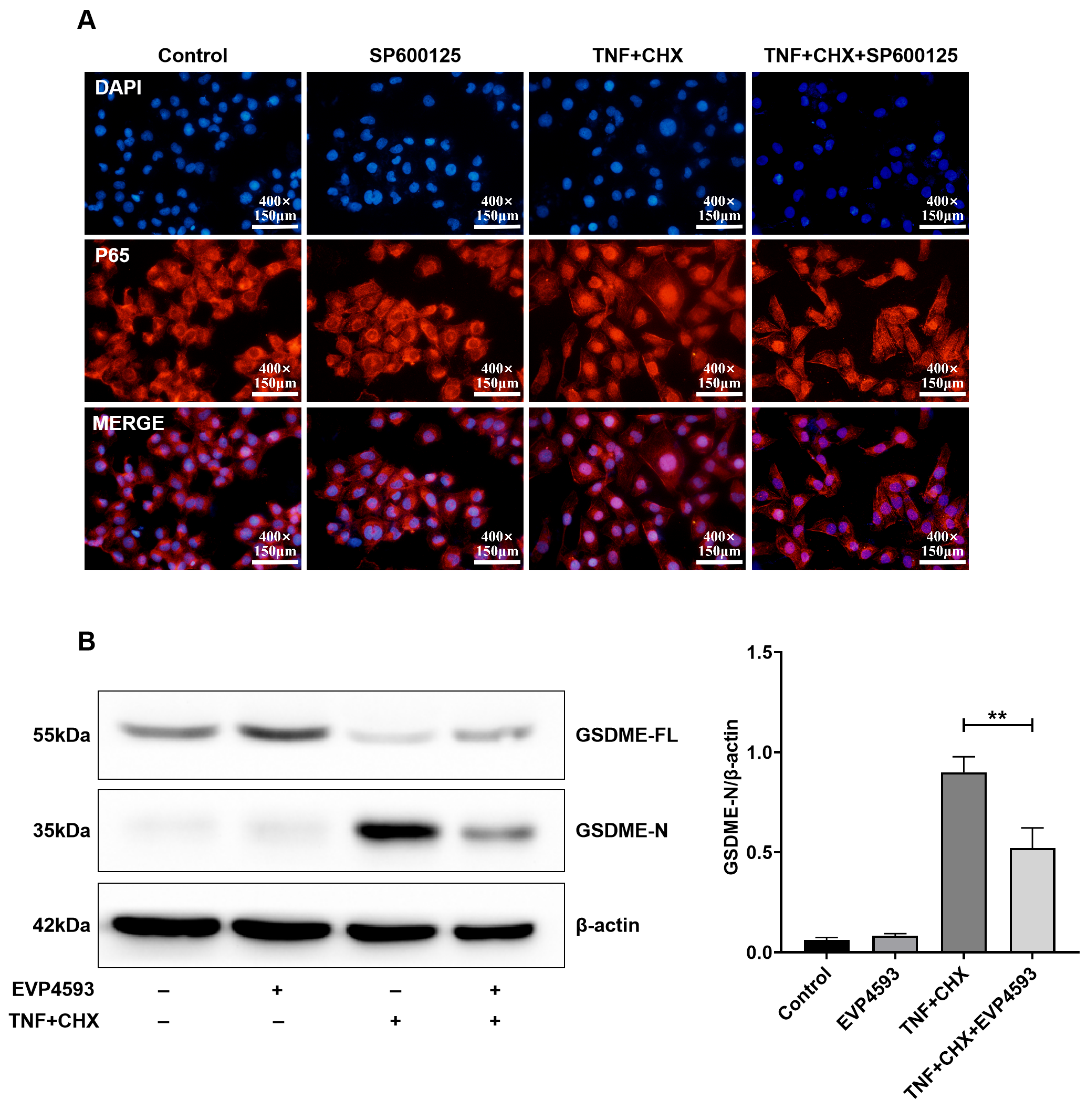

Supplement: Supplementary file 2 — Supplementary Figure 1 [file 41420_2022_848_MOESM2_ESM.tif]

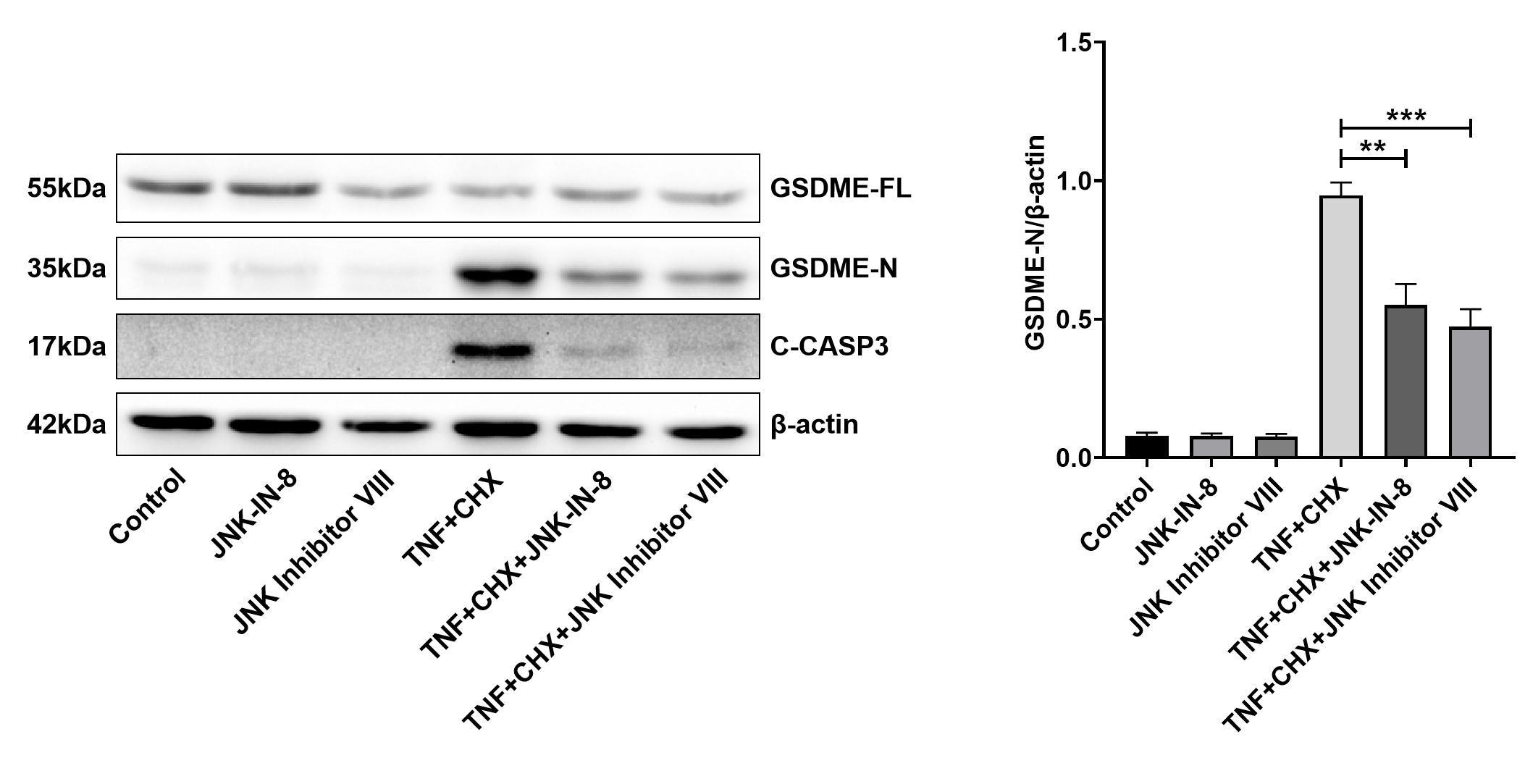

Supplement: Supplementary file 3 — Supplementary Figure 2 [file 41420_2022_848_MOESM3_ESM.tif]
