# Supplementary material for: Blocking GSDME-mediated pyroptosis in renal tubular epithelial cells alleviates disease activity in lupus mice
Source: Cell Death Discov. 2022 Mar 12;8:113. doi: 10.1038/s41420-022-00848-2 (PMC8918340; doi:10.1038/s41420-022-00848-2)
Supplement: Supplementary file 5 — AJE Editing Certificate [file 41420_2022_848_MOESM5_ESM.pdf]

Editing Certificate

This document certifies that the manuscript

Blocking GSDME-mediated pyroptosis in renal tubular epithelial cells alleviates disease activity in lupus mice

prepared by the authors

Guihu Luo, Yi He, Fangyuan Yang, Zeqing Zhai, Jiaochan Han, Lili Zhuang, Yanang Zhang, Yehao Li, Rui Song, Xiaoqing Luo, Jianheng Liang, Erwei Sun

was edited for proper English language, grammar, punctuation, spelling, and overall style by one or more of the highly qualified native English speaking editors at AJE.

This certificate was issued on **November 30, 2021** and may be verified on the AJE website using the verification code **5E9D-3AE9-FB5F-56A1-EBAP** .

Neither the research content nor the authors' intentions were altered in any way during the editing process. Documents receiving this certification should be English-ready for publication; however, the author has the ability to accept or reject our suggestions and changes. To verify the final AJE edited version, please visit our verification page at aje.com/certificate. If you have any questions or concerns about this edited document, please contact AJE at support@aje.com.

AJE provides a range of editing, translation, and manuscript services for researchers and publishers around the world. For more information about our company, services, and partner discounts, please visit aje.com.
